# The predictive value of abnormal P-wave axis for the detection of incident atrial fibrillation: A systematic review with meta-analysis

**DOI:** 10.1371/journal.pone.0278527

**Published:** 2022-12-01

**Authors:** Rahul Kumar Chattopadhyay, Panagiota Anna Chousou, Trisha Mukherjee, Peter John Pugh, Vassilios S. Vassiliou

**Affiliations:** 1 Norwich Medical School University of East Anglia, Norwich, United Kingdom; 2 Addenbrookes Hospital Cambridge University Hospitals NHS Foundation Trust, Cambridge, United Kingdom; 3 Norfolk and Norwich University Hospitals NHS Foundation Trust, Norwich, United Kingdom; 4 Institute of Continuing Education, University of Cambridge, Cambridge, United Kingdom; Chulalongkorn University, THAILAND

## Abstract

**Introduction:**

There is growing interest in the prediction of incident atrial fibrillation (AF). The 12-lead electrocardiogram (ECG) has been a particularly rich target for possible prediction strategies.

**Purpose:**

The P-wave axis is an ECG parameter that reflects the dominant vector of atrial depolarisation and is usually 0° -75°. There is a large body of literature suggesting that AF reflects structural and conduction abnormalities of the atria, and thus the P-wave axis may represent a sensitive parameter to detect such changes.

**Methods:**

A systematic review and meta-analysis of published literature associating abnormal P-wave axis and the development of incident AF was performed. Electronic databases were systematically searched from inception to October 2021. A random-effects model with generic inverse variance weights was utilised to pool the most adjusted effect measure from each paper. A funnel plot was used to assess publication bias.

**Results:**

After excluding duplicate studies, 568 studies were screened. A total of eleven studies were identified that associated an abnormal P-wave axis with the subsequent detection of AF. The eight studies that considered abnormal P-wave axis as being <0° or >75° were pooled for meta-analysis. In the pooled studies a total of 78,222 patients were included with 5656 cases of incident atrial fibrillation identified. The meta-analysis of the studies suggested that an abnormal P-wave axis was associated with a pooled risk ratio of 2.12 (95% CI 1.49 to 3.01) for the detection of incident atrial fibrillation.

**Conclusion:**

This comprehensive systematic review and meta-analysis, indicates the positive association of abnormal P wave axis and future detection of AF. Utilisation of abnormal P-wave axis, alongside other parameters, may allow clinicians to better risk-stratify individuals at increased risk of AF, and thus identify those who may benefit most from prolonged cardiac monitoring or targeted anticoagulation.

## Introduction

The frontal P-wave axis reflects the dominant vector of atrial depolarisation, and can be calculated by analysis of the P-wave in the limb leads on a 12-lead electrocardiogram (ECG) with reference to the hexaxial system [1]. Its association with respiratory disease, and in particular emphysema, was previously well explored [2–4]. More recently it has become a topic of interest with regards to its predictive potential for the detection of incident atrial fibrillation (AF). The combination of significant morbidity and mortality associated with AF, combined with the safety profile of direct oral anticoagulants, has resulted in a drive to identify predictors of AF to optimise pharmacotherapy and minimise morbidity and mortality.

The normal P-wave axis is defined by angles between 0° and 75°. An abnormal P-wave axis may either be right-wardly displaced, which is typically defined as > 75°, or left-wardly displaced, defined as <0°. The former may be seen a variety of conditions, including right atrial enlargement, emphysema without right atrial enlargement and conduction abnormalities including ectopic atrial rhythms. Leftward deviation can be seen in left atrial enlargement as well as atrial conduction abnormalities.

There have been several large cohort studies looking at the relationship between abnormal P-wave axis and detection of future incident AF, whilst there have also been smaller case-control studies considering specific patient sub-groups. The pathophysiological correlate for this relationship remains an area of active investigation. Gross structural changes in the atria such as dilatation are associated both with a change in P-wave axis and AF, [5] but variations in axis may represent more subtle changes such as fibrosis of the atrial conduction tissue, prior to AF development.

This systematic review and meta-analysis aims to provide a summary of the literature pertaining to the potential role of P-wave axis as a predictor for future AF detection.

## Methods

The Preferred Reporting Items for Systematic Reviews and Meta-Analysis (PRISMA) [6] and Meta-analysis of Observational Studies in Epidemiology (MOOSE) statements [7] were utilised to guide this systematic review and meta-analysis

### Search strategy

The electronic databases PUBMED, EMBASE, Web of Science and Cochrane were systematically searched using the key words “P-wave axis” and “P wave axis” from inception to October 2021. A subsequent “snowball” search was performed to identify further studies, by examining the reference lists of included studies.

### Study selection

An initial titular analysis, followed by abstract and full text screening was performed. The following criteria were utilised a) any observational study including case-control, nested case-control, retrospective and prospective cohort studies; b) studies pertaining to the relationship between abnormal P-wave axis and AF; c) exclusion of baseline AF; d) full-text availability; e) human studies. For the purposes of this review, atrial flutter was considered alongside AF. Abstracts and non-peer reviewed studies were excluded.

### Data extraction

Data was extracted from the identified studies using a pre-specified electronic collection form. The data extracted included study location, population, population size, mean population age, population gender distribution, the number of individuals who developed incident AF, how incident AF was diagnosed, the follow up time, the definition of abnormal P-wave axis utilised, methodology for P-wave axis calculation, sample exclusion criteria, reported hazard (HR) or odds ratios (OR) and confounders utilised in multivariable assessment. The main outcome of interest was the development of incident AF.

### Statistical analysis

For the purposes of the meta-analysis, where an effect size with 95% confidence interval from a multivariable analysis was available, this was used. Where a study quoted a hazard ratio, this was converted to an odds ratio, using methodology defined in the Cochrane Handbook for Systematic Reviews of Interventions [8]. A random-effects model with generic inverse variance weights was utilised to pool the effect measures. Statistical heterogeneity was assessed using the I^2^ statistic. A two-tailed P-value <0.05 was considered statistically significant. A funnel plot was used to identify possible publication bias. All statistical analyses were conducted using the Review Manager (RevMan) software (version 5.3. Copenhagen: The Nordic Cochrane Centre, The Cochrane Collaboration, 2014).

The quality of each study was assessed utilising the Newcastle-Ottawa scale (NOS) [9].

## Results

**Fig 1:**
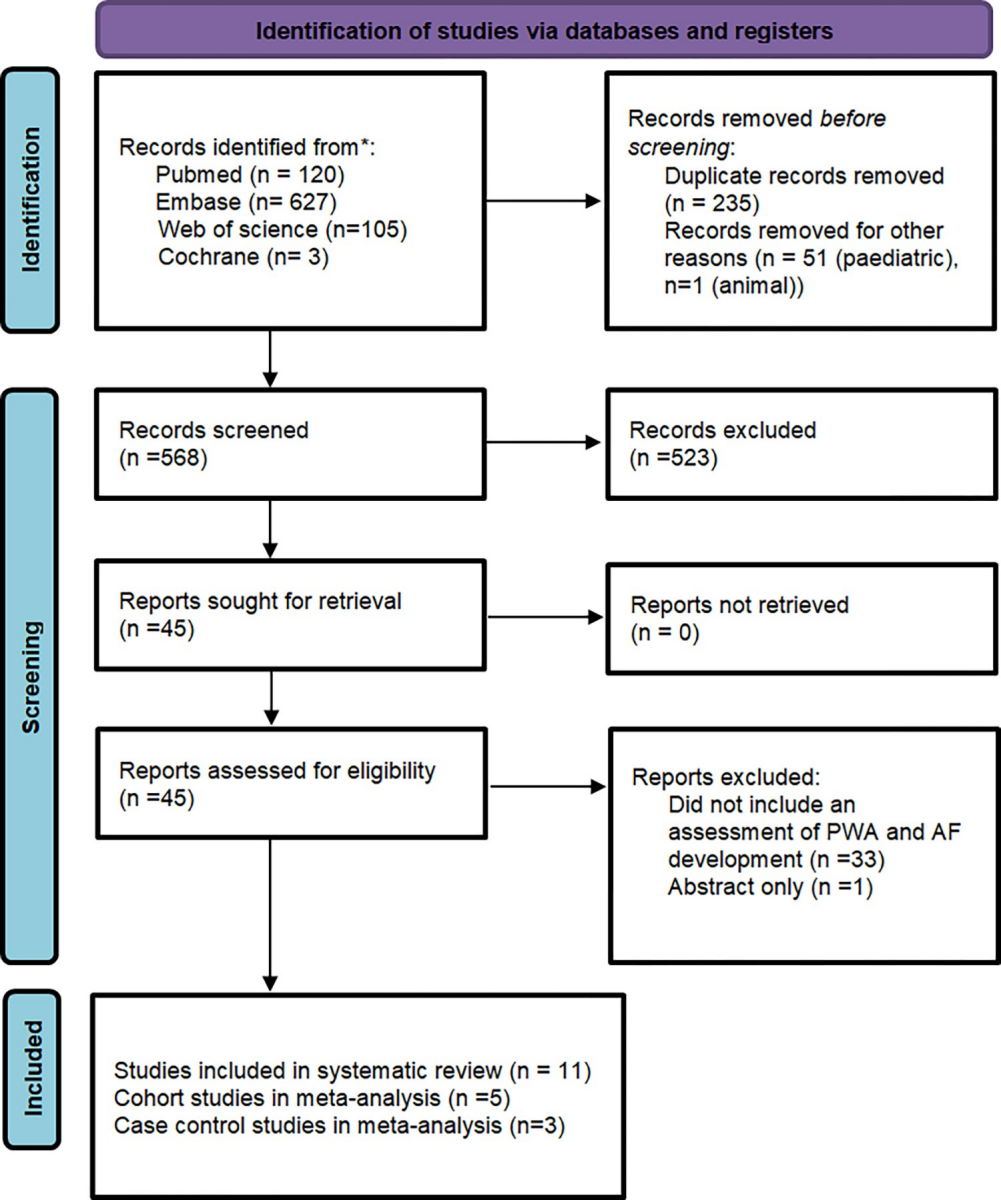
PRISMA diagram for systematic review and meta-analysis.

A total of 855 studies were screened across the different databases. Following removal of duplicates, animal and paediatric studies, 568 studies were further examined. A total of eleven studies were identified that related an abnormal p-wave axis with the subsequent detection of atrial fibrillation, as outlined in Fig 1. Further manual searching of the identified studies citations did not unveil any further studies for inclusion. The quality of each paper was assessed with the NOS as shown in S1 Fig. The baseline characteristics and study details of each study are presented in Table 1. Studies that considered abnormal P-wave axis as being <0° or >75° were pooled for meta-analysis.

**Table 1:** Characteristics of studies included in systematic review ACCORD (Action to Control Cardiovascular Risk in Diabetes); AF (atrial fibrillation); ARIC (Atherosclerosis Risk in Communities); BMI (body mass index); CHD (coronary heart disease); CHS (Cardiovascular Health Study); CI (Confidence interval); CHARGE-AF (Cohorts for Heart and Aging Research in Genomic Epidemiology); DM (diabetes mellitus); ECG (electrocardiogram); ESUS (Embolic Stroke of uncertain source); HDL (High density lipoproteins); HF (heart failure); HR (Hazard ratio); ILR (Implantable loop recorder); LAE (left atrial enlargement); LBBB (left bundle branch block); LVH (left ventricular hypertrophy); MA (meta-analysis); MVA (multivariable analysis); NOS (Newcastle-Ottawa score); OR (Odds ratio); PAC (premature atrial complexes); pAF (paroxysmal atrial fibrillation); PVC (premature ventricular complexes); PWA (P-wave axis); PPM (permanent pacemaker); PWTF (P-wave terminal force); s (seconds); SBP (systolic blood pressure); USA (United states of America).

Author	Year	Location	Study population	Sample size	Mean Age (years)	Male (%)	AF patients	Diagnostic method for AF	Follow up time	Normal PWA	NOS	Methodology	Exclusions	PWA	Normal PWA	Abnormal PWA	Effect size	Converted OR	Variables in MVA
**Yildirim et al.**	2019	Turkey	Hospital patients	140	51.41 ± 15.58 (sinus) 65.87 ± 10.80 (AF)	59 (42.1%)	70	ECG	13 months	0–75	5	Case control	No sinus ECGs in pAF patients	Not specified	108	32	Adjusted OR 5.96 (95% CI 1.89–18.78)	-	PR duration, P-wave dispersion, P-wave max time, P-wave peak time, PWTF
**Perez et al.**	2009	USA	General population	42751	55.8 ± 15 (sinus) 67.5 ± 10.5 (AF)	36723 (85.1%)	1050	ECG	Mean 5.3 years	0–75	8	Cohort	Prevalent AF	Computerised analysis	40980	1771	Adjusted HR 1.9 (95% CI 1.6–2.4)	OR 1.92	Age, male sex, PR interval >200ms, PAC, P_max_>120 ms; P_index_ >35msec; LAE, PVC, LBBB, LVH
**Hayashi et al.**	2013	Japan	P-pulmonale	591	56.4 ± 14.8	382 (64.6%)	61	ECG	60.8±71.4 months (non-AF) 45.1±64.8 months (AF)	<74	-	Case-control	Non-sinus baseline rhythm	Not specified	-	-	Adjusted HR 2.55 (95% CI 1.20–5.41)	Not included in MA	Age, gender, presence of various diseases, ECG variables
**Rangel et al.**	2017	USA	CHS	4274	Not specified	1734 (40.6%)	1274	ECG + discharge data	Median 12.1 years	0–75	8	Cohort	Missing data, prevalent AF	Computerised + averaging	3124	1150	Adjusted HR 1.17 (95% CI 1.03–1.33)	OR 1.17	Age, sex, race, education, income, smoking, DM, CHD, stroke, HF, heart rate, SBP, BMI, total cholesterol, HDL cholesterol, antihypertensive medications, aspirin, statins
**Lehtonen et al.**	2017	Finland	Community-dwelling Finns	5667	51.5 ± 14.1	2577 (45.5%)	423	Discharge or death certificates	Mean 11.9+/- 2.9 years	0–75	8	Cohort	Missing data, prevalent AF	Automated	5244	423	Adjusted OR 1.59 (95% CI 1.19–2.13)	-	Age, sex, BMI, current smoking, chronotropic medication, DM, CHD, HF, heart rate
**Maheshwari et al.**	2017	USA	ARIC	15078	54.2 ± 5.7	7311 (48.5%)	2618	ECG, discharge data, death certificates	Mean 20.3 years	0–75	8	Cohort	Missing data, prevalent AF	Manual	11252	3826	Adjusted HR 2.34 (95% CI 2.12–2.58)	OR 2.66	CHARGE-AF variables
**Cho et al.**	1997	S Korea	Bradycardia PPM—VVI	80	58.9 ± 11.4	28 (35%)	13	ECG	25.7 ± 2.5 months	0–90	-	Case-control	-	Not specified	72	8	Non-significant	Not included in MA	-
Acampa et al. [24]	2018	Italy	Cryptogenic stroke	222	78 ± 72	108 (48.6%)	44	ECG + 7 day monitoring	7 days	0–75	5	Case-control	-	Computerised analysis	189	33	Adjusted OR 3.3 (95% CI 1.49–7.35)	-	Age, atrial size, ECG parameters
**Dhaliwal et al.**	2020	USA	ACCORD	8965	62.5 ± 6.5 (Normal PWA) 64.9 ± 7.1 (Abnormal PWA)	5068 (61.2%) 437 (63.9%)	145	ECG (baseline, 2 year, end)	Mean 7.7 years	0–75	6	Case control	Unclear P-wave, prevalent AF	Automated	8281	684	Adjusted HR 2.69 (95% CI, 1.77–3.97)	OR 2.72	Age, sex, race
**Li et al.**	2021	China	ESUS	181	63.0 ± 12.3	128 (70.7%)	14	ILR (70) or clinically	Median 2.1 years IQR 1.4–2.8	0–75	6	Cohort	-	Manual	170	11	OR 1.21 (95% CI 0.14–10.18)	-	-
**Kreimer et al.**	2021	Germany	ILR–any indication	366	62 ± 16	191 (52.2%)	75	ILR detection >30s	627+/-409 days	0–75	-	Cohort	AF, no interrogation reports	Manual	-	-	Not significant	Not included in MA	Age, ECG parameters

Considering the three studies that were not included in the meta-analysis–Cho et al. utilised an upper P-wave axis limit of 90°; Hayashi et al. reported AF prediction with P-wave axis less than 74° in patients with P-pulmonale; Kreimer et al. did not report an overall numerical risk statistic for abnormal P-wave axis.

**Fig 2:**
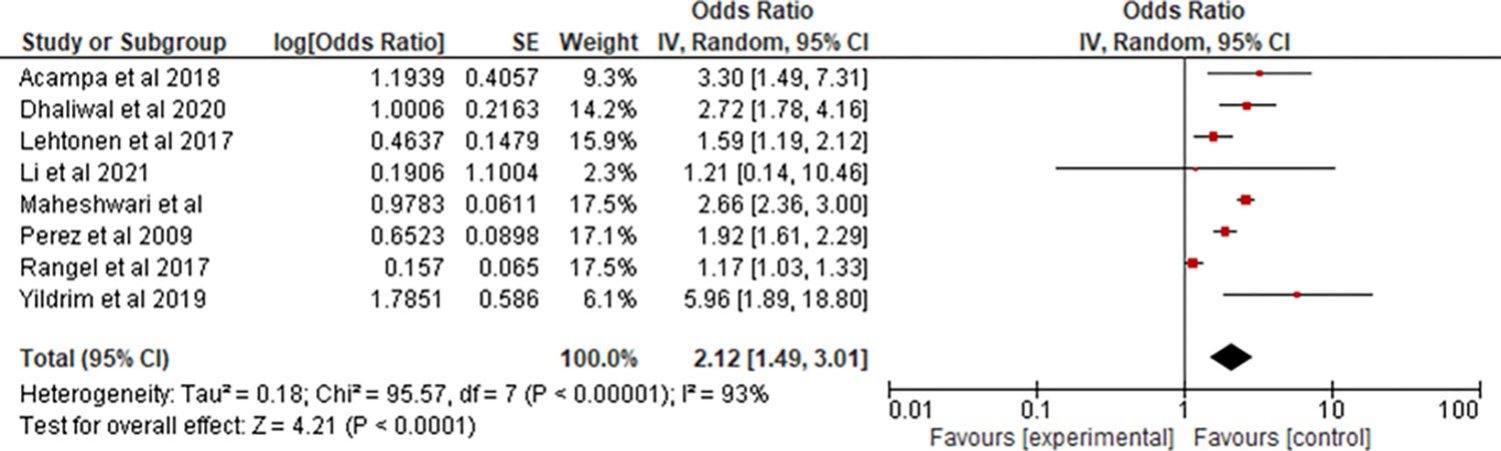
Forest plot of studies included in meta-analysis.

In the pooled studies a total of 78327 patients were included with at least 5656 cases of incident AF identified. Seven of the eight pooled studies quoted an effect size from a multivariable analysis, which was thus utilised, with one study using univariate analysis. The meta-analysis of the studies suggested that an abnormal P-wave axis was associated with a pooled effect size of 2.10 (95% confidence interval (CI) 1.48 to 2.72) for the detection of incident AF (Fig 2). Heterogeneity was high, with a I^2^ value of 92%. The effect of this was reduced by utilising -a random effects model. To identify the source of heterogeneity, a sensitivity analysis was performed by removing one study at a time (S2 Fig), which demonstrated that the Rangel et al. [10] study was responsible for a significant degree of the identified heterogeneity, with a remaining association of 2.29 (95% CI 1.81 to 2.92). Following the exclusion of the Rangel et al.(10) the I^2^ value remained high at 70%.

**Fig 3:**
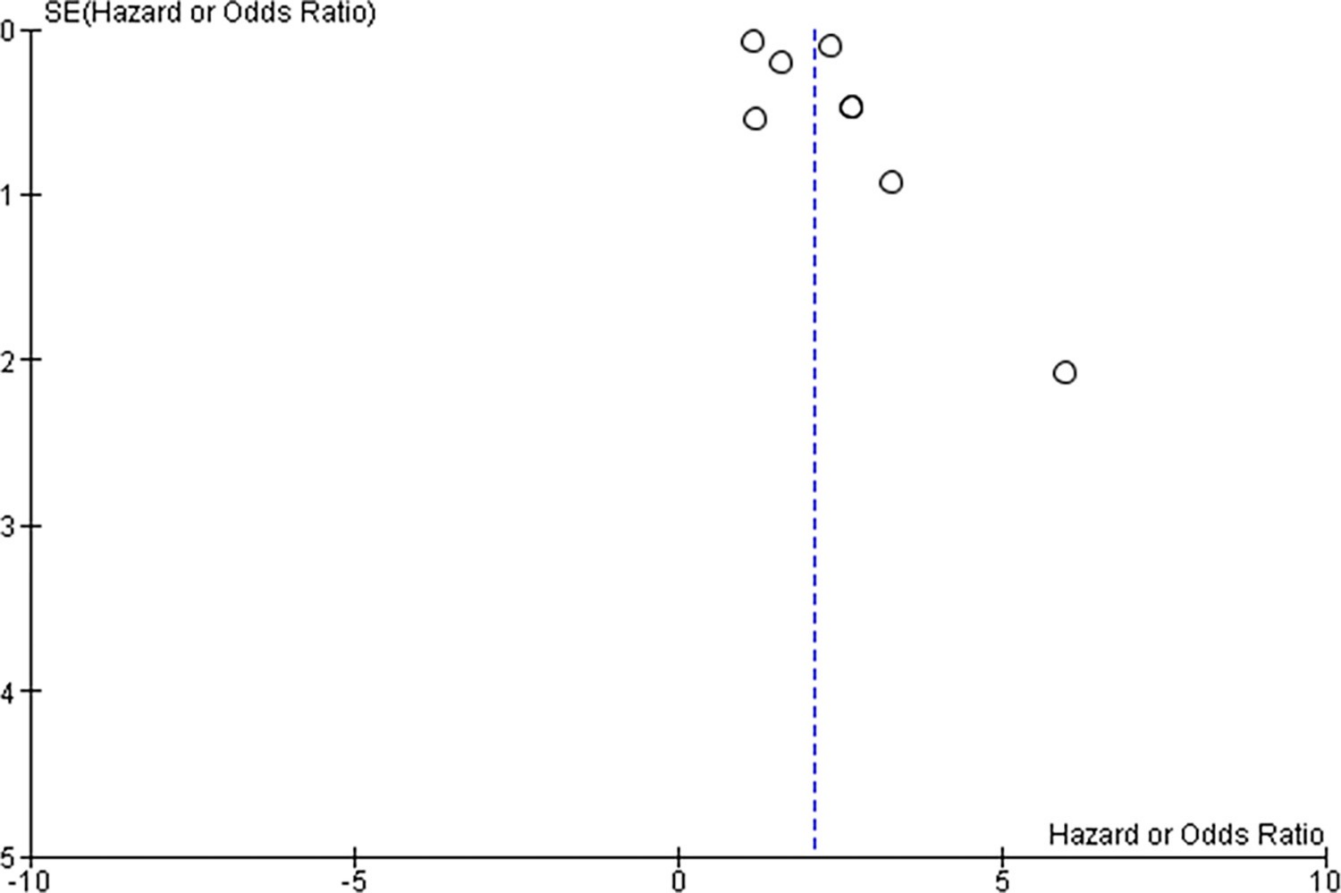
Funnel plot of included studies.

A funnel plot looking for possible publication bias in the field is shown in Fig 3. Whilst confounded by the small number of studies included in the meta-analysis, this suggests possible publication bias for the positive studies. However, it is also possible that the risk associated with P-wave axis abnormalities is so strong that all studies show the association.

## Discussion

### Summary of results

This is the first systematic review and meta-analysis of the association of abnormal P-wave axis with AF.

The results of this meta-analysis suggest that an abnormal P-wave axis is a positive predictor for the detection of incident AF. This was apparent in all but one of the eight studies that were appropriate for meta-analysis.

Considering the large cohort studies, abnormal P-wave axes were shown to have an association with the development of future AF in the Action to Control Cardiovascular Risk in Diabetes (ACCORD), Cardiovascular Health Study (CHS), Atherosclerosis Risk in Communities (ARIC) and Perez’s Californian populations, over an average follow up time of between 5.3 and 20.3 years.

In terms of studies that did not identify an association, both Li et al. and Kreimer et al. failed to identify a statistically significant association in smaller cohorts [11, 12], the former in embolic strokes of unclear source (ESUS) and the latter in an unselected population of patients undergoing implantable loop recorder (ILR) insertion. Similarly Cho et al.’s [13] prospective case control study of patients undergoing bradycardia indication pacemaker implantation did not demonstrate an association between abnormal P-wave axis and AF, although this was in the context of a different definition of abnormal P-wave axis of 0° to 90°.

Lehtonen et al.’s [14] large assessment of community dwelling Finnish individual failed to identify any association between abnormal leftward or rightward P-wave axis and incident AF, individually, although when the results are combined a significant risk ratio for abnormal P-wave axis is demonstrated

Hayashi et al. [15] did look at P-wave axis as a predictor for incident AF, but this was specifically in the population of patients with P-pulmonale which is typically associated with right atrial enlargement. This is reflected in the fact that both the AF group and the non-AF group had similar mean (standard deviation) P-wave axis of 73.2° (8.0°) and 75.4° (8.4°) respectively. Thus their finding that a P-wave axis <74° had a HR of 2.55 (95%CI 1.20–5.41) was particularly interesting, and is likely related to the fact that the normal P-wave axis in p-pulmonale is rightward, and thus non-right ward axes suggest further atrial abnormalities beyond that causes by axis changes due to lung changes impacting cardiac position.

### AF detection

Only two of the studies made use of continuous ILR monitoring to detect incident AF, [11, 12] whilst all of the others utilised a combination of sporadic ECGs, patient notes or death certificates. Even though Li et al. utilised ILR based monitoring, less than half of the cohort had ILR implantation due to the need for patient based co-payment. Given the paroxysmal nature of AF, it is plausible that there is under-detection of incident AF in these studies. However, it is worthy of note that ILR studies would not easily be able to mirror the follow up duration seen in the other studies due to them having a maximal battery life of 4 years.

### Reproducibility of P-wave axis

A criticism of other P-wave indices has been their susceptibility to lead related changes and poor short-term reproducibility [16]. Since the P-wave axis is wholly dependent on the limb leads, it is more resolute to lead position than markers such as P-wave terminal force. Indeed the short term reliability and stability was quantitatively demonstrated in 63 individuals within the Atherosclerosis Risk in Communities (ARIC) study, where between two ECGs done between two weeks, the intraclass correlation coefficient was 0.78, which was better than that seen for both P-wave duration and P-wave terminal force [17].

### Alternative ECG predictors of AF

The P-wave axis, is just one of a number of different ECG based parameters that have been studied as a possible predictor of the development of future AF. Generally the most well studied parameters are P-wave related and include measures such as P-wave duration, PR segment duration and measures of inter-atrial block [18]. More novel parameters such as the P-wave terminal force have shown promise as possible predictors of AF [19], although there are concerns regarding its reproducibility [20].

Ventricular parameters, such as ECG defined left ventricular hypertrophy, QT interval and QRS duration have also been investigated [18]. Perhaps unsurprisingly, these do not tend to be as strong predictors as the atrial parameters.

There is likely to be overlap between the predictive role of these different ECG parameters, and larger, carefully designed studies are necessary to disentangle the effect of individual parameters. The strength of the P-wave axis as an ECG predictor lies in its reproducibility, and the fact that it is frequently reported on a standard 12-lead ECG.

### The significance of P-wave axis association

This finding is important, as it implies that patients with an abnormal P-wave axis, are at an increased risk of AF development. With the number of populations that this finding was demonstrated in, the consistency of this in meta-analysis, and the fact that P-wave axis is automatically reported on many digital ECG reports, this may represent an easy to identify predictor of future AF. The fact that this result persisted in multivariable analyses suggests that it may act synergistically with other predictors, such as hypertension, hyperthyroidism and obesity for AF. Ultimately, combining a large cohort study with methods of continuous monitoring such as an ILR, would help further establish the role of P-wave axis, although the follow up period would be limited to the battery life of the device.

### The clinical implications of P-wave axis as a predictor of AF

An abnormal P-wave axis may be used alongside other established parameters, to help refine AF prediction. Identifying individuals who are susceptible to the development of incident AF, is beneficial across different populations.

In the general population, identifying such individuals can help to target continuous monitoring methods such as ILR implantation, improving the yield of AF screening approaches. Moreover, with the improved understanding of lifestyle factors that propagate atrial fibrillation, such as obesity [21] and excess alcohol intake [22], aggressive lifestyle modification can be targeted to try and reduce the risk of AF development.

In the ESUS population, this group of individuals may provide a more promising target for empirical anticoagulation. NAVIGATE-ESUS [23] demonstrated that bind anticoagulation of all patients does not reduce stroke recurrence, but is associated with worsened morbidity.

### Limitations

This meta-analysis is composed of data from observational studies. As such heterogeneity was high which is typical in the diverse cohorts of patients seen in observation studies. Measures were taken however to minimise this effect including utilisation of a random effects model and a sensitivity analysis. Furthermore, both the follow up period between the studies, and the method of AF detection varied significantly.

Finally, we were not able to include in the meta-analysis all the studies identified in the systematic review which might allowed a more accurate estimation of the risk associated with abnormal p wave axis. As mentioned in the results section, three studies could not be included in the meta-analysis. Two papers used different values to define an abnormal P-wave axis. Hayashi et al. used less than 74°, which is abnormal in their population of P-pulmonale patients, but would be considered normal in the general population. The Cho et al. paper used values greater than 90°, and therefore could not be included in our meta-analysis, as all of the other papers included considered P-waves axes of 75° to 90° abnormal. Kreimer et al. did not provide a numerical value for their effect size.

## Conclusion

This comprehensive systematic review and meta-analysis, including 78222 patients from eight studies, indicates the positive association of abnormal P-wave axis and future detection of AF. It thus confirms a two-fold risk of abnormal p-wave axis independent of other established predictive factors such as hypertension, age and abnormal systolic heart function. Utilisation of abnormal P-wave axis, can allow clinicians to better risk-stratify individuals and provide more frequent monitoring for AF in individuals at high risk.

## Supporting information

S1 Checklist PRISMA 2020 checklist. (DOCX)

S1 Fig Newcastle-Ottawa scale quality scores for each study included in the meta-analysis. (TIF)

S2 Fig Forest plot of sensitivity analysis. Sensitivity analysis demonstrating continued association of P-wave analysis despite exclusion of Rangel et al.
(TIF)
