# Supplementary figures and images for: The predictive value of abnormal P-wave axis for the detection of incident atrial fibrillation: A systematic review with meta-analysis
Source: PLoS One. 2022 Dec 1;17(12):e0278527. doi: 10.1371/journal.pone.0278527 (PMC9714955; doi:10.1371/journal.pone.0278527)

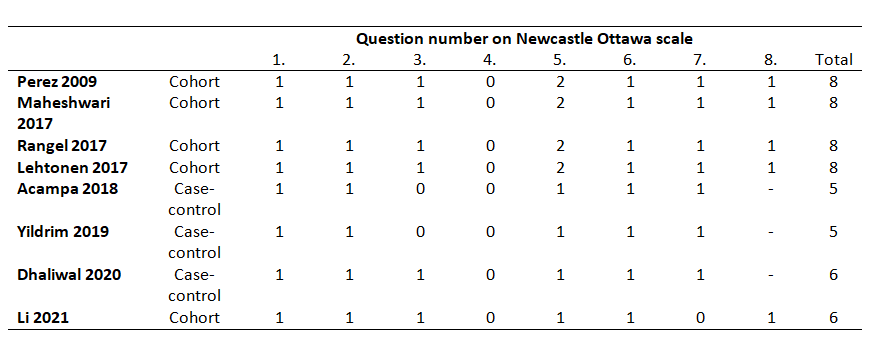

Supplement: S1 Fig — (TIF) [file pone.0278527.s002.tif]

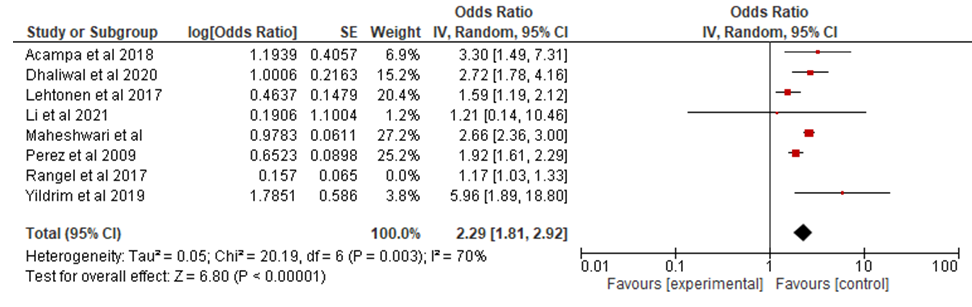

Supplement: S2 Fig — Sensitivity analysis demonstrating continued association of P-wave analysis despite exclusion of Rangel et al. (TIF) [file pone.0278527.s003.tif]
